# Low-complexity regions within protein sequences have position-dependent roles

**DOI:** 10.1186/1752-0509-4-43

**Published:** 2010-04-13

**Authors:** Alain Coletta, John W Pinney, David Y Weiss Solís, James Marsh, Steve R Pettifer, Teresa K Attwood

**Affiliations:** 1 Faculty of Life Sciences, University of Manchester, Manchester M13 9PL, UK; 2 School of Computer Science, University of Manchester, Manchester M13 9PL, UK; 3 Switch Laboratory, Department of Applied Biological Sciences, Vrije Universiteit Brussel, 1050, Belgium; 4 Centre for Bioinformatics, Division of Molecular Biosciences, Imperial College London, London SW7 2AZ, UK; 5 Institute of Interdisciplinary Research (IRIBHM), School of Medicine, Free University of Brussels, 1070 Brussels, Belgium; 6 IRIDIA-CoDE, Université Libre de Bruxelles, Ave. F. Roosevelt 50, 1050 Brussels, Belgium

## Abstract

**Background:**

Regions of protein sequences with biased amino acid composition (so-called Low-Complexity Regions (LCRs)) are abundant in the protein universe. A number of studies have revealed that i) these regions show significant divergence across protein families; ii) the genetic mechanisms from which they arise lends them remarkable degrees of compositional plasticity. They have therefore proved difficult to compare using conventional sequence analysis techniques, and functions remain to be elucidated for most of them. Here we undertake a systematic investigation of LCRs in order to explore their possible functional significance, placed in the particular context of Protein-Protein Interaction (PPI) networks and Gene Ontology (GO)-term analysis.

**Results:**

In keeping with previous results, we found that LCR-containing proteins tend to have more binding partners across different PPI networks than proteins that have no LCRs. More specifically, our study suggests i) that LCRs are preferentially positioned towards the protein sequence extremities and, in contrast with centrally-located LCRs, such terminal LCRs show a correlation between their lengths and degrees of connectivity, and ii) that centrally-located LCRs are enriched with transcription-related GO terms, while terminal LCRs are enriched with translation and stress response-related terms.

**Conclusions:**

Our results suggest not only that LCRs may be involved in flexible binding associated with specific functions, but also that their positions within a sequence may be important in determining both their binding properties and their biological roles.

## Background

Low-complexity regions (LCRs) in protein sequences are regions containing little diversity in their amino acid composition. The degree of diversity they exhibit may vary, ranging from regions comprising few different amino acids, to those comprising just one, the amino acid positions within these regions being either loosely clustered, irregularly spaced, or periodic [1]. This work defines LCRs computationally as an amino acid sequence with low information content (see methods). Therefore, simple repetitive sequences such as tandem amino acid repeats form part of the LCR dataset discussed here.

LCRs are common in protein sequences, but precise measures of their abundance are difficult to ascertain. One of the problems is that the degrees of stringency applied by different detection methods differ, leading to different estimates of the numbers of LCRs in the same dataset. Importantly also, our knowledge of the protein universe has changed dramatically during the last 15 years, as protein sequence repositories have become engorged with the outputs of high-throughput sequencing projects. Protein sequence databases have thus grown enormously (both in terms of the numbers of sequences they contain and in terms of the numbers of organisms represented), and estimates of the numbers of LCRs they contain have changed accordingly: *e.g*., the proportion of proteins in the Swiss-Prot database that contain LCRs has changed from 56%, in 1993 (V-26.0) [2], to 12% in the current version of UniProt (V-54.0) [3]. Notwithstanding their abundance in protein sequences, LCRs are largely under-represented in the Protein Data Bank (PDB) [4,5], presumably because most of the proteins containing LCRs do not readily crystallise. Despite this lack of structural information, LCRs are believed to play pivotal roles across a wide range of biological functions [6-8], some of whose mechanisms have been extensively documented, although the proposed functional models remain unverified [8-10].

### Low-complexity regions evolve rapidly through recombination events

LCRs are known to evolve rapidly, sometimes via mitotic replication slippage, or, more often, via meiotic recombination events [11]. Highly dynamic diversification of these regions, and high levels of inter-species variation and polymorphism, suggest that newly generated and expanded LCRs are, in most cases, structurally and functionally neutral, with a high probability of fixation [12], thus generating novel material that could enable rapid functional expansions. Moxon and co-workers suggested that repeat formation is a common source of genetic variation among prokaryotes to generate novel surface antigens and adapt to fast evolving environments [7,13]. This source of variability may also compensate for longer generation times in eukaryotes, which have higher proportions of LCRs [11] and it has been suggested that expansions and contractions of tandem repeats constitute a large source of phenotypic variation [6].

### Hub proteins contain more LCRs than non-hub proteins

While some LCRs are known to play important structural roles by acquiring strong static conformations [14], others have been associated with intrinsically unstructured proteins [15,16]. The flexible nature of regions lacking well-defined folding structures is thought to be responsible for their versatile binding capabilities; this flexibility could allow these regions to bind several different targets [17]. In their recent study on yeast protein-protein interactions (PPIs), Ekman and co-workers noted that the highly connected 'hub' proteins contain an increased fraction with LCRs compared to non-hub proteins [12]. They suggested that disordered regions are particularly important for flexible binding and could act as flexible linkers between globular protein domains. Here, we set out to investigate whether proteins with LCRs tend to have larger numbers of binding partners across a range of high confidence PPI datasets. We then examined whether proteins with LCRs positioned at their sequence extremities show differences in connectivity compared to proteins with LCRs positioned in central regions, and if the number of protein binding partners is related to LCR length. Finally, we functionally categorised both terminal-LCR and central-LCR groups using Gene Ontology [18] (GO)-term enrichment analysis.

## Results and Discussion

In this study, we used data from the yeast *Saccharomyces cerevisiae*, as this was the most comprehensive for our purposes. We used four PPI datasets (Table 1): three high-confidence datasets (FYI [19], HC [20], and DIP-verified (DIPv) [21]), where each interaction is confirmed by more than one detection method, and a lower-confidence but more extensive dataset (BioGrid [22]) containing all interactions reported to date.

**Table 1:** Nodes and edges in each PPI dataset

	BioGrid	HC	FYI	DIPv
Number of nodes	4884	2977	2545	2278
Number of edges	37989	9203	5953	5373

The FYI [19] is generated as the union of: Yeast two-hybrid experiments [23-25], datasets produced from affinity purification and mass spectrometry screens [26,27], one dataset produced from *in silico *computational prediction methods [28], the physical protein-protein interactions, excluding interactions from genome-scale experiments, from the Munich Information Center for Protein Sequences (MIPS) [29] Comprehensive Yeast Genome Database (CYGD) dataset [30], and finally, the CYGD protein complexes published in the literature (called LC for **L**iterature **C**urated data). The resulting union is then filtered keeping only interactions observed at least twice by different detection methods.

The HC PPI dataset [20] is also a join of multiple interaction datasets, were the minimal criterion for inclusion is that relevant interactions must be independently reported at least twice. This differs from the FYI in that two independent reports can come from two datasets using identical detection methods. HC uses LC data from five major PPI databases - BIND [31], BioGrid [22], DIP [32], MINT [33] and MIPS [29], and interactions detected from affinity purification and mass spectrometry screens [34,35]. The DIPv dataset [21] is a computationally verified core of the DIP dataset [32], which is a database of experimentally verified interactions determined by several techniques (such as genome-wide two hybrid screen-including results from [23] and [24]-, immunoprecipitation, affinity binding, and antibody blockage).

The DIPv core was computed using two methods: the **E**xpression **P**rofile **R**eliability (EPR) index, and the **P**aralogous **V**erification **M**ethod (PVM). EPR compares RNA expression profiles of potentially interactive proteins against expression profiles of known interacting, and non-interacting pairs of proteins. PVM measures the likelihood that two proteins interact by measuring interactions between their paralogues. We refer to this dataset as DIP-verified (DIPv).

*S. cerevisiae *is also amongst the most well-annotated genomes, making it ideal for functional analysis using the Gene Ontology [18]. In agreement with previous estimates [36], our LCR-detection method (see Methods) found that of 6, 165 *S. cerevisiae *proteins documented in UniProt, 1; 306 contained LCRs. Of these, 929 contain a unique LCR; to simplify the analyses presented, this study deals only with proteins containing a single LCR.

### Proteins containing LCRs tend to have more interactions than those without

**Figure 1:**
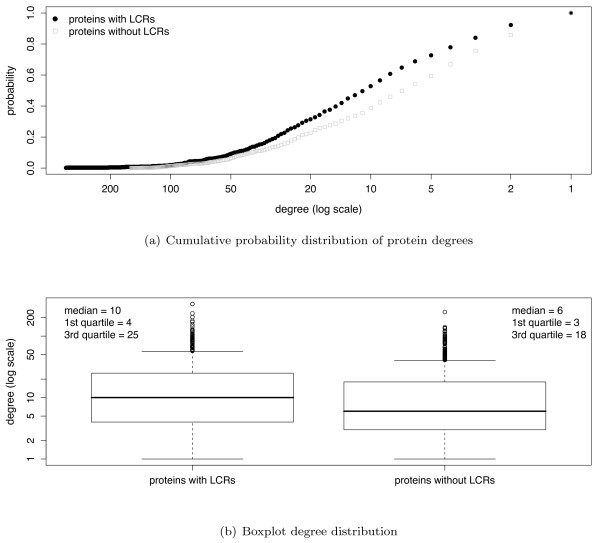
**Degree distributions comparison between proteins with and without LCRs**. Degree distributions of proteins with and without LCRs in the BioGrid dataset show proteins with LCRs have more connections than proteins without LCRs. See Table 2 for Wilcoxon-Mann-Whitney *p*-values for this and the other datasets.

We considered two subsets of yeast proteins: those with one LCR and those without LCRs. The degree (*i.e*., connectivity) distributions of both subsets were computed for the four PPI network datasets used in this study. By way of illustration, the degree distributions in the BioGrid network are shown in Figure 1.

Comparing the degree distributions using the Wilcoxon-Mann-Whitney test shows that proteins containing LCRs appear to have more protein interactions than proteins without LCRs in all four PPI datasets (all networks having *p *< 0.05, see Table 2).

**Table 2:** Degree distributions comparison between protein with and without LCRs.

dataset	BioGrid	HC	FYI	DIPv
*p*-value	1.58 × 10^-13^	3.63 × 10^-04^	0.002	0.021

Wilcoxon-Mann-Whitney test *p*-values obtained from comparing degree distributions from proteins with and without LCRs across the four different PPI datasets.

### LCR locations are biased towards protein sequence extremities

**Figure 2:**
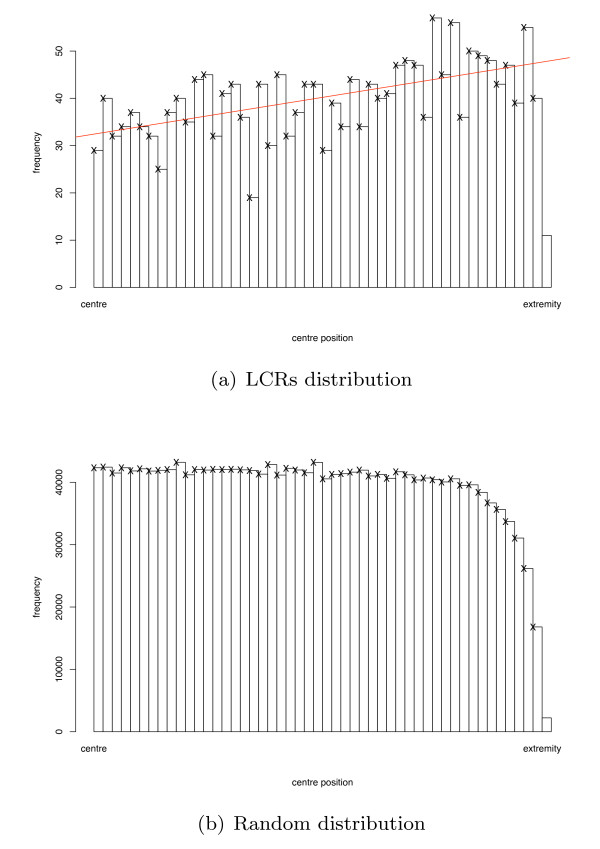
**Distribution of folded LCR centre positions**. Comparison of normalised and randomly re-arranged LCR centre positions in *S. cerevisiae*. The Kolmogorov-Smirnov test confirms that these two distributions are significantly different (*p*-value = 7.6 × 10^-6^).

To investigate whether LCR locations are positionally significant, we examined whether LCRs occur randomly within protein sequences. We located the centre positions of LCRs on a continuous scale ranging from the centre to the extremities of the protein sequence by recording their normalised centre positions and folding the resulting distribution in half. We compared the actual distribution of their centres to an empirical null distribution derived from a random model (see Figure 2 and Additional file 1: Figure S1). This null distribution was constructed by removing the LCR from each protein sequence, then repeatedly re-inserting it at random start positions (see Additional file 2: Figure S2). The empirical null distribution is approximately uniform near the centre of the protein sequence and decreases sharply near the sequence extremities. By contrast, the observed frequency of real LCRs increases steadily from the centre to the near extremities (Figure 2(a)). The Kolmogorov-Smirnov test confirms that natural LCR positions do not follow our computed random distribution (*p*-value = 7.6 × 10^-6^), implying that the position of the LCR within the protein sequence may be of relevance to its function.

### Terminal LCRs are more connected than central LCRs and show length-connectivity dependence

**Figure 3:**
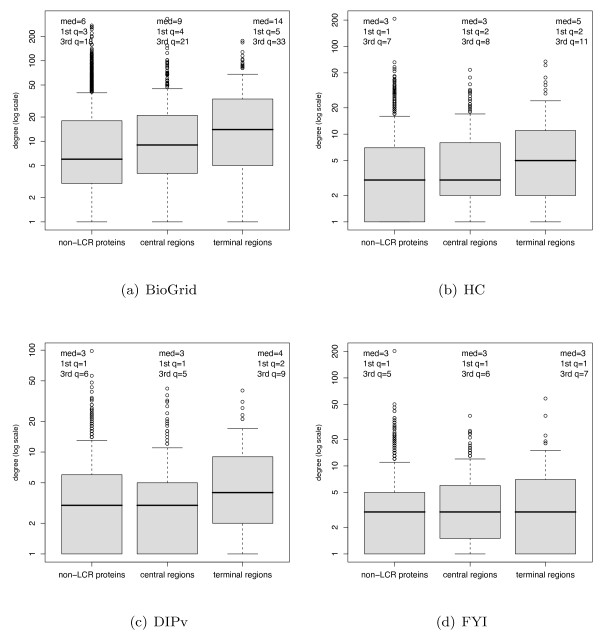
**Degree distribution comparisons**. Boxplot representations comparing degree distributions of t-LCRs, c-LCRs, and proteins without LCRs. Table 4 shows Wilcoxon-Mann-Whitney *p*-values resulting from comparing their degree distributions.

**Figure 4:**
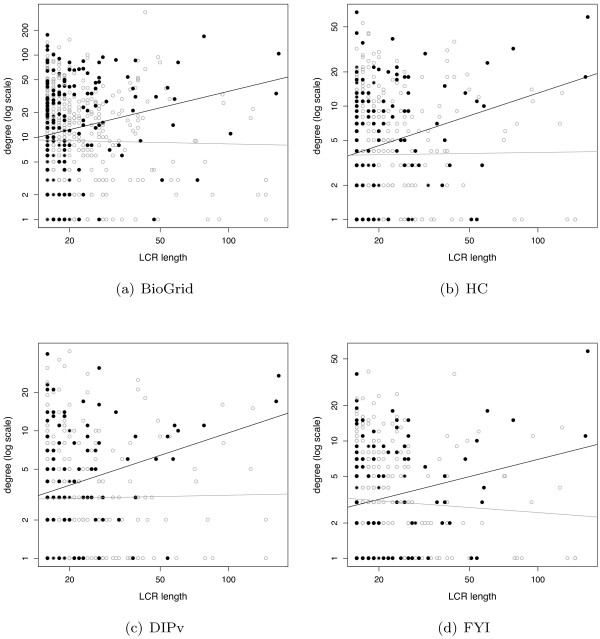
**LCR length versus protein degree**. Scatterplots show the relationship between length and protein degree for t-LCRs (in black) and c-LCRs (in gray) in four different PPI networks. The associated *p*-values and *r*^2^-values for linear regression are shown in Table 5.

To further characterise the properties of LCRs in our study, we tested whether protein connectivity is related to LCR position within the sequence. We defined two sub-populations of LCRs: terminal LCRs (t-LCRs), occurring near the sequence extremities, and central LCRs (c-LCRs), positioned far from the sequence extremities. To ensure that t-LCRs are truly positioned at the sequence termini, they were defined as regions starting or ending at no more than 25 amino acids from either sequence extremity; c-LCRs, on the other hand, were defined as regions positioned at least 50 amino acids from either sequence extremity. The number of c-LCRs and t-LCRs found in the different PPI datasets are shown in Table 3. To investigate the properties of our two LCR populations, we first compared the degree distributions of t-LCRs, c-LCRs and non-LCR proteins. Results presented in Figure 3 show that proteins with t-LCRs are more connected than proteins with c-LCRs in three out of four networks (Table 4). t-LCRs clearly tend to be more connected than non-LCR proteins, with significant differences across all four networks. c-LCRs also appear to have higher degrees than non-LCRs, with *p *< 0.05 in three out of four networks. We then examined whether LCR length is related to protein degree in each population. Figure 4 shows that the length of t-LCRs is positively correlated to their protein degree, while there is no sign of such correlation amongst the population of c-LCRs. *r*^2 ^values are small owing to the large scatter in protein degrees, which is presumably caused by a combination of the uncertainties in PPI network data and the fact that proteins may also bind via interfaces that are independent of LCRs. Notwithstanding these effects, the *p*-values associated with each linear regression line show that proteins with t-LCRs have significant correlations between LCR length and degree across all four PPI networks studied (Table 5).

**Table 3:** Number of t-LCRs and c-LCRs found across the four PPI datasets.

	BioGrid	HC	FYI	DIPv
t-LCRs	183	135	123	109
c-LCRs	493	349	299	263

**Table 4:** Degree distributions comparison between protein with c-LCRs, t-LCRs, and proteins without LCRs.

	t-LCRs/c-LCRs	c-LCRs/*non-LCRs*	t-LCRs/*non-LCRs*
BioGrid *p*-value	0.001	1.94 × 10^-07^	1.54 × 10^-10^
HC *p*-value	0.005	0.031	6.88 × 10^-04^
DIPv *p*-value	0.01	0.471	0.001
FYI *p*-value	0.587	0.044	0.051

Wilcoxon-Mann-Whitney test *p*-values were calculated to compare the degree distributions of proteins with t-LCRs, c-LCRs, and without LCRs across the four different PPI datasets.

**Table 5:** Correlation results (LCR length versus protein degree).

	*central LCRs*		*terminal LCRs*	
	*p*-value	*r*^2^-value	*p*-value	*r*^2^-value
BioGrid	0.672	3.66 × 10^-04^	0.004	0.043
HC	0.837	1.22 × 10^-04^	0.004	0.06
DIPv	0.792	2.68 × 10^-04^	0.006	0.069
FYI	0.263	0.004	0.019	0.045

The table shows statistics for the regression lines plotted in Figure 4. The *p*-values show the probability that LCR length is uncorrelated with protein degree, as calculated by an *F*-test.

### GO analysis shows that terminal and central LCRs have different biological roles

We then performed GO-term enrichment analyses for the set of all LCR proteins, and for the c-LCR and t-LCRs subsets, in order to gain insights into their respective functions. Results show that the set of proteins with LCRs is enriched for functions related to the regulation of gene expression. Furthermore, the analysis suggests that t-LCRs and c-LCRs have distinct cellular roles. The first analysis compared all proteins with LCRs against the entire *S. cerevisiae *proteome as background, and showed enrichments for ten GO terms at a false-discovery rate (*q*-value) threshold of 0:01. Table 6 gives a detailed description of these terms, their frequencies, *p*-values and *q*-values. This ensemble of GO term enrichments suggests that LCRs have a tendency to find roles in transcription, transcription regulation and translation. Interestingly, the term 'nucleic acid binding' suggests that the binding capabilities of LCR proteins may not be restricted to protein-protein interactions. The same analysis was performed with t-LCRs and c-LCRs separately, and revealed t-LCR enrichments for 32 GO terms and c-LCR enrichments for 22 GO terms under the same *q*-value threshold (Table 7). Proteins with t-LCRs are important to stress response, translation and transport processes and are enriched in protein complexes, while proteins with c-LCRs are important in transcription and transcription regulation processes and are enriched for kinase functions. Although these groups share common and functionally related GO terms, the fact that our somewhat arbitrary division of LCRs into central and terminal subsets results in lower *q*-values (and hence more significant GO term enrichments) than in the complete LCR population supports the hypothesis that LCR location is directly implicated in protein function.

**Table 6:** GO term enrichments for all LCRs.

Frequencies					
Genes	Background	*p*-value	*q*-value	GO term ID	definition
49	147	3.89 × 10^-06^	0.003	(P)GO:0006950	**response to stress**
117	518	4.40 × 10^-05^	0.017	(P)GO:0006350	**transcription**
41	133	1.03 × 10^-04^	0.026	(P)GO:0006468	**protein amino acid phosphorylation**
11	15	2.22 × 10^-04^	0.042	(P)GO:0006414	**translational elongation**
105	490	6.08 × 10^-04^	0.092	(P)GO:0006355	**regulation of transcription, DNA-dependent**

73	294	1.25 × 10^-04^	0.054	(F)GO:0003676	**nucleic acid binding**

51	189	2.59 × 10^-04^	0.066	(C)GO:0005730	**nucleolus**
30	93	4.58 × 10^-04^	0.066	(C)GO:0009277	**fungal-type cell wall**
344	1946	6.27 × 10^-04^	0.066	(C)GO:0005634	**nucleus**
22	63	0.001	0.088	(C)GO:0005934	**cellular bud tip**

GO term enrichments from proteins with LCRs compared to the entire *S. cerevisiae *proteome. Frequencies represent the number of proteins annotated by a given term, *p*-values are calculated using Fisher's exact test, *q*-values are calculated using Benjamini & Hochberg's FDR method.

**Table 7:** GO term enrichments for central and terminal LCRs.

Terminal LCRs					
**Frequencies**					

**Genes**	**Background**	***p*-value**	***q*-values**	**GO term ID**	**definition**
22	147	1.09 × 10^-10^	2.76 × 10^-08^	(P)GO:0006950	**response to stress**
28	418	3.64 × 10^-06^	4.62 × 10^-04^	(P)GO:0006412	**translation**
6	15	8.55 × 10^-06^	7.24 × 10^-04^	(P)GO:0006414	**translational elongation**
5	10	2.19 × 10^-05^	0.001	(P)GO:0006616	**SRP-dependent cotranslational protein targeting to membrane, translocation**
5	26	8.99 × 10^-04^	0.046	(P)GO:0006893	**Golgi to plasma membrane transport**

13	114	1.37 × 10^-05^	0.002	(F)GO:0016887	**ATPase activity**
16	202	9.10 × 10^-05^	0.005	(F)GO:0003735	**structural constituent of ribosome**
5	33	0.002	0.087	(F)GO:0004175	**endopeptidase activity**
30	703	0.004	0.087	(F)GO:0000166	**nucleotide binding**
4	24	0.005	0.087	(F)GO:0005484	**SNAP receptor activity**
5	40	0.005	0.087	(F)GO:0003743	**translation initiation factor activity**
3	12	0.006	0.087	(F)GO:0003746	**translation elongation factor activity**
2	3	0.006	0.087	(F)GO:0019904	**protein domain specific binding**
7	85	0.008	0.092	(F)GO:0051082	**unfolded protein binding**
4	28	0.008	0.092	(F)GO:0003688	**DNA replication origin binding**
2	4	0.009	0.093	(F)GO:0008353	**RNA polymerase subunit kinase activity**
21	290	2.40 × 10^-05^	0.003	(C)GO:0005840	**ribosome**
5	14	7.83 × 10^-05^	0.006	(C)GO:0015935	**small ribosomal subunit**
19	284	1.63 × 10^-04^	0.008	(C)GO:0030529	**ribonucleoprotein complex**
6	43	0.001	0.038	(C)GO:0043234	**protein complex**
4	16	0.001	0.038	(C)GO:0000502	**proteasome complex**
3	9	0.003	0.051	(C)GO:0000786	**nucleosome**
3	9	0.003	0.051	(C)GO:0000788	**nuclear nucleosome**
3	9	0.003	0.051	(C)GO:0005852	**eukaryotic translation initiation factor 3 complex**
6	53	0.003	0.052	(C)GO:0022627	**cytosolic small ribosomal subunit**
3	10	0.004	0.052	(C)GO:0043614	**multi-eIF complex**
2	3	0.006	0.065	(C)GO:0034099	**luminal surveillance complex**
2	3	0.006	0.065	(C)GO:0030133	**transport vesicle**
2	3	0.006	0.065	(C)GO:0031201	**SNARE complex**
3	14	0.008	0.082	(C)GO:0005667	**transcription factor complex**
6	68	0.010	0.096	(C)GO:0030686	**90S preribosome**
11	189	0.011	0.098	(C)GO:0005730	**nucleolus**

**Central LCRs**					

**Frequencies**					

**Genes**	**Background**	***p*-value**	***q*-value**	**GO term ID**	**definition**

27	133	3.03 × 10^-09^	1.40 × 10^-06^	(P)GO:0006468	**protein amino acid phosphorylation**
50	518	4.38 × 10^-06^	0.001	(P)GO:0006350	**transcription**
45	490	4.52 × 10^-05^	0.007	(P)GO:0006355	**regulation of transcription, DNA-dependent**
7	18	9.81 × 10^-05^	0.011	(P)GO:0006378	**mRNA polyadenylation**
24	123	4.64 × 10^-08^	1.03 × 10^-05^	(F)GO:0004674	**protein serine/threonine kinase activity**
66	703	2.18 × 10^-07^	1.68 × 10^-05^	(F)GO:0000166	**nucleotide binding**
23	125	2.28 × 10^-07^	1.68 × 10^-05^	(F)GO:0004672	**protein kinase activity**
55	577	1.88 × 10^-06^	1.04 × 10^-04^	(F)GO:0005524	**ATP binding**
15	90	8.39 × 10^-05^	0.004	(F)GO:0004386	**helicase activity**
23	204	2.94 × 10^-04^	0.011	(F)GO:0016301	**kinase activity**
28	294	8.31 × 10^-04^	0.026	(F)GO:0003676	**nucleic acid binding**
10	61	0.001	0.036	(F)GO:0008026	**ATP-dependent helicase activity**
6	22	0.001	0.036	(F)GO:0004407	**histone deacetylase activity**
3	4	0.003	0.066	(F)GO:0004708	**MAP kinase kinase activity**
4	11	0.004	0.077	(F)GO:0005543	**phospholipid binding**
5	19	0.004	0.077	(F)GO:0016566	**specific transcriptional repressor activity**
15	63	2.04 × 10^-06^	3.39 × 10^-04^	(C)GO:0005934	**cellular bud tip**
132	1946	4.07 × 10^-06^	3.39 × 10^-04^	(C)GO:0005634	**nucleus**
26	189	5.24 × 10^-06^	3.39 × 10^-04^	(C)GO:0005730	**nucleolus**
5	9	2.89 × 10^-04^	0.014	(C)GO:0005849	**mRNA cleavage factor complex**
5	12	7.97 × 10^-04^	0.031	(C)GO:0000508	**Rpd3L complex**
16	129	9.96 × 10^-04^	0.032	(C)GO:0005935	**cellular bud neck**

GO term enrichments from proteins with c-LCRs and t-LCRs compared to the complete set of proteins in *S. cerevisiae*.

## Conclusions

Our results show that LCRs are preferentially located towards sequence extremities, and that proteins with LCRs in their sequence extremities have more protein binding partners than proteins with LCRs in their central regions. Furthermore, we have shown the length of LCRs to be positively correlated with the number of binding partners, but only in the sequence extremities. While t-LCRs can extend free from the rest of the protein structure, c-LCRs are likely to be surrounded by protein globular domains, thus limiting their flexibility and accessibility, and therefore the number of different proteins to which they can mediate binding. By contrast, if t-LCRs themselves tend to act as promiscuous interfaces for protein binding, this would explain our observation that proteins with longer t-LCR regions have a tendency towards a higher number of protein binding partners. Examining the list of over-represented GO terms in Table 7, we hypothesise that t-LCRs play major roles in low-specificity biological events that involve large protein complexes. Protein chaperones, for example, which play a major role in stress response, have low-specificity binding properties due to the large variety of partners they bind to assist conformational search towards global energy minima [37,38]. Translation and translation elongation are also events requiring low-specificity interactions, involving a crowded protein machinery that operates on the entire proteome. Finally, molecular transport could also be considered to fall within this category, with large protein complexes moving a wide variety of cargos across the cell.

Although some c-LCRs might still be expected to act as flexible linkers, there is evidence that they may also act as direct binding interfaces, albeit with more restricted promiscuity than t-LCRs. Kim and co-workers [39] found that disordered regions could function as interfaces with a limited number of binding partners, particularly in the context of phosphorylation cascades in signalling pathways, where proteins tend to contain both a structured kinase domain and an unstructured kinase-binding domain. Indeed, regions of protein disorder are already known to be implicated in signalling as phosphorylation sites [40]. Our GO analysis finds protein kinase functions to be over-represented only for the set of central LCRs, and not those located at the termini, hence could be considered to be consistent with the existence of a specific set of binding partners for each signalling protein. The set of c-LCR proteins is also enriched with other biological processes that, although still 'promiscuous' in the sense that they have multiple binding partners, need to be much more specific than the translation, folding, and transport processes observed for the t-LCRs. Transcription regulation events, for example, limit the number of proteins present simultaneously [41]. Binding events in polyadenylation processes are also relatively specific and do not involve crowded protein machineries.

In their recent study on protein-protein interactions, Ekman and co-workers noted that hub proteins (those with a large number of interacting partners) are more often multi-domain proteins and contain more disordered regions compared to non-hubs. This observations led them to stress that the disordered regions serve as linkers between domains, in addition to their more commonly reported role in flexible or rapidly reversible binding [12]. Our proteome-wide results show that these two LCR functional roles are distinct and depend on the location of the LCRs within the protein sequence: their role in flexible and rapidly reversible binding is preferentially mediated by LCRs located in the terminal regions of proteins while their role as linkers between protein domains is preferentially mediated by centrally located LCRs.

These results, together with the other differences in GO enrichment discussed above, suggest that the functions of the low-complexity regions of a protein are related in a fundamental manner to their positions within the sequence.

## Methods

### Implementation of the LCRs detection algorithm

We used Shannon's entropy, *H*, as the measure to detect LCRs, as it is the most well-accepted measure of complexity in biological sequences [36](1)

where *P*_*i *_represents the fraction of the amino acid at position *i *within the string of interest. The difficulty is that LCRs vary widely in length and position, and it is not reasonable to use the same complexity threshold for every sequence length. Therefore, we scanned the whole proteome for window lengths, varying from 16 to 300 amino acids, to compute the distributions of entropy values (10^12 ^measurements). This provided a background to test whether a single entropy value would be sufficiently extreme to be considered an LCR. For each window, *w*, the frequency density of the calculated Shannon entropy values is represented by a histogram *f*_*w*_(*H*). Let *A*_*w *_be a cumulative density function, the area underneath this histogram:(2)

Given (2), a low-complexity threshold value, *t*_*w*_, is calculated for every window, *w*, as the entropy limit holding 0.5% of the cumulative distribution function such that:(3)

We define a low-complexity region as any window of length *w *with an entropy value smaller than *t*_*w*_. Entropy distributions for every window length are highly skewed, with a bell-shaped curve at high entropy values and a very long and thin tail extending toward the low entropy values where LCRs are located (see Additional file 3: Figure S3). Given that all entropy distributions for any window length have a similar shape, a single cut-off point selects the same proportion of low-entropy regions, enriched LCRs, regardless of window length.

A very conservative threshold was sought to exclude non-LCR. Visual inspection determined that a threshold corresponding to 0.5% of the area under the distribution curve only included the portion of the curve where the flat tail, containing the LCRs, was located. A very conservative threshold was chosen to have a stringent cut-off and exclude non-LCRs.

#### Selecting LCRs in protein sequences

Entropy values from different window lengths have comparable distribution shapes (Additional files: Figure S3 and S4), and are therefore standardised for comparison. Entropy value distributions from longer regions have smaller standard deviations and greater means. By contrast, distributions from shorter regions have greater standard deviations and smaller means. Overlapping LCRs are common during the detection process; in order to compare entropy scores from LCRs of different length, the implemented algorithm computes a standardised *Z*-score for each detected LCR.(4)

where *H *is the entropy, *μ*_*w *_the mean, and *σ*_*w *_the standard deviation of *f*_*w*_(*H*). If multiple LCRs overlap, only the region with the highest *Z*-score is retained. All detected regions can be accessed and queried through the UTOPIA User Interface [42].

### PPI datasets

Analyses were cross-validated over four PPI datasets: three high-confidence datasets (HC [20], DIPv [21] and FYI [19]) and one, potentially of lower-confidence, but much larger set of interactions (BioGrid [22]). Although the comparison of the three different high-confidence PPI datasets, FYI, HC and DIPv, showed a much greater overlap than previous datasets [43], there were still large numbers of differences between them (Additional file 4: Figure S5). Therefore, inter-study validation using the three high-confidence and the BioGrid PPI datasets was performed to ensure robust results. To ensure that only information relevant to protein-protein interactions was obtained from the BioGrid network, it was first stripped of all non-physical interactions, as described in [44]. To determine whether LCRs are equally distributed across PPI datasets, the study also investigated the distribution of LCRs within the different PPI datasets. Results showed that the three high-confidence networks were similarly enriched in LCRs (approximately 19% of their entries contain LCRs, see Additional file 5: Table S1). These enrichments in the high-confidence networks support the idea that these regions are highly interactive.

### Measurements of region positions in protein sequences, correlations, and comparison of degree distributions

We defined the position of an LCR as the coordinate of the LCR's centre within the protein sequence in which it occurs. We then divided this coordinate by the length of the protein to express it on a normalised scale between 0 and 1. The result is an LCR position metric comparable across LCRs of varying lengths within proteins of varying lengths. t-LCRs were defined as regions starting or ending at no more than 25 amino acids from either sequence extremity, c-LCRs as regions starting or ending at least 50 amino acids from either sequence extremity. Correlation *p*-values and regression lines were computed using the linear model function implemented in the R statistics package. Degree distributions were compared using the Wilcoxon Mann-Whitney test, also implemented in the R statistics package.

### GO-term enrichment analyses

GO-term enrichment *p*-values were calculated using Fisher's exact test [45], and transformed to *q*-values using Benjamini and Hochberg's multiple testing correction method [46], as implemented in the R statistics package, version 2.7.

## Authors' contributions

AC, SRP and TKA conceived the study. AC, JWP, DW and TKA designed the research. AC carried out the research. AC, JWP, DW and JM analysed the results. AC, JWP, TKA and DW wrote the paper. All authors read and approved the final manuscript.

## Supplementary Material

Additional file 1 **Figure S1: LCR distributions in PPI datasets**. PPI datasets overlap between the HC, DIPv, FYI and BioGrid datasets, and the distribution of LCRs among them. Click here for file

Additional file 2 **Figure S2: Mean and standard deviation from UniProt entropy distributions**. The entropy distributions mean grows asymptotically towards the *H*_*max *_value as the window regions increase and sequences within them approach random states. The entropy distributions standard deviation decreases as longer sequences become more homogeneous. Click here for file

Additional file 3 **Figure S3: Computing random LCR positions**. Method to compute random LCR positions. The same process is repeated for each LCR in *S. cerevisiae*: LCRs (shown in red) are extracted from their corresponding protein sequence and re-inserted randomly 1000 times. Each time, the normalised centre position is included into the random distribution. Click here for file

Additional file 4 **Figure S4: LCR centre positions distribution**. Distributions of LCR centre positions and randomly replaced LCR centre positions. The random distribution extremities show the expected frequency decrease, while the original distribution on top, appears to be enriched with extremity LCRs. Click here for file

Additional file 5 **Table S1: LCR distributions in PPI datasets**. LCRs are approximately equally distributed across the high-confidence datasets (HC, FYI and DIPv). Enrichment is defined as (*Observed - Expected*)/*Expected*. Click here for file

## References

[B1] DePristo M Zilversmit M Hartl D On the abundance, amino acid composition, and evolutionary dynamics of low-complexity regions in proteins Gene 2006 378 19 30 10.1016/j.gene.2006.03.023 16806741

[B2] Wootton J Federhen S Statistics of local complexity in amino acid sequences and sequence databases Computers chem 1993 17 2 149 163 10.1016/0097-8485(93)85006-X

[B3] UniProt-Consortium The universal protein resource (UniProt) Nucleic Acids Research 2008 36 D190 5 10.1093/nar/gkm895 18045787 PMC2238893

[B4] Huntley M Golding G Simple sequences are rare in the Protein Data Bank Proteins 2002 48 134 140 10.1002/prot.10150 12012345

[B5] Berman M Westbrook J Feng Z Gilliland G Bhat T Weissig H Shindyalov I Bourne P The protein data bank Nuc Acids Res 2000 28 235 242 10.1093/nar/28.1.235 PMC102472 10592235

[B6] Fondon J Garner H Molecular origins of rapid and continuous morphological evolution P Natl Acad Sci Usa 2004 101 52 18058 18063 10.1073/pnas.0408118101 PMC539791 15596718

[B7] Verstrepen K Jansen A Lewitter F Fink G Intragenic tandem repeats generate functional variability Nat Genet 2005 37 9 986 90 10.1038/ng1618 16086015 PMC1462868

[B8] Phatnani H Greenleaf A Phosphorylation and functions of the RNA polymerase II CTD Genes Dev 2006 20 2922 2936 10.1101/gad.1477006 17079683

[B9] Zagon I Verderame M McLaughlin P The biology of the opioid growth factor receptor (OGFr) Brain Res Brain Res Rev 2002 38 351 376 10.1016/S0165-0173(01)00160-6 11890982

[B10] Wanker E Sun Y Savitz A Meyer D Functional characterization of the 180-kD ribosome receptor in vivo J Cell Biol 1995 130 29 39 10.1083/jcb.130.1.29 7790375 PMC2120505

[B11] Marcotte E Pellegrini M Yeates T Eisenberg D A Census of Protein Repeats Journal of Molecular Biology 1999 293 151 160 10.1006/jmbi.1999.3136 10512723

[B12] D Ekman SL Bjorklund A Elofsson A What properties characterize the hub proteins of the protein-protein interaction network of the protein-protein interaction network of Saccharomyces cerevisiae? Genome Biology 2006 7 6 R45 10.1186/gb-2006-7-6-r45 16780599 PMC1779539

[B13] Moxon E Rainey P Nowak M Lenski R Adaptive evolution of highly mutable loci in pathogenic bacteria Current Biology 1994 4 24 33 10.1016/S0960-9822(00)00005-1 7922307

[B14] Tatham A Shewry P Elastomeric proteins: biological roles, structures and mechanisms Trends Biochem Sci 2000 25 11 567 571 10.1016/S0968-0004(00)01670-4 11084370

[B15] Tompa P Intrinsically unstructured proteins Trends Biochem Sci 2002 27 10 527 533 10.1016/S0968-0004(02)02169-2 12368089

[B16] Dunker A Obradovic Z Romero P Garner E Intrinsic protein disorder in complete genomes Genome Informatics 2000 11 161 171 11700597

[B17] Dyson H Wright P Intrinsically unstructured proteins and their functions Nature Reviews Molecular Cell Biology 2005 6 197 208 10.1038/nrm1589 15738986

[B18] Ashburner M Ball C Blake J Botstein D Butler H Cherry J Davis A Dolinski K Dwight S Eppig J Harris M Hill D Issel-Tarver L Kasarskis A Lewis S Matese J Richardson J Ringwald M Rubin G Sherlock G Gene ontology: tool for the unification of biology. The Gene Ontology Consortium Nat Genet 2000 25 25 29 10.1038/75556 10802651 PMC3037419

[B19] Bertin N Simonis N Dupuy D Cusick M Han J Fraser H Roth F Vidal M Confirmation of organized modularity in the yeast interactome Plos Biol 2007 5 6 e153 10.1371/journal.pbio.0050153 17564493 PMC1892830

[B20] Batada N Reguly T Breitkreutz A Boucher L Breitkreutz B Hurst L Tyers M Still stratus not altocumulus: further evidence against the date/party hub distinction Plos Biol 2007 5 6 e154 10.1371/journal.pbio.0050154 17564494 PMC1892831

[B21] Deane C Salwinski L Xenarios I Eisenberg D Protein Interactions Two Methods for Assessment of the Reliability of High Throughput Observations Molecular and Cellular Proteomics 2002 1 349 356 10.1074/mcp.M100037-MCP200 12118076

[B22] Breitkreutz B Stark C Reguly T Boucher L Breitkreutz A Livstone M Oughtred R Lackner D Bahler J Wood V Dolinski K Tyers M The BioGRID Interaction Database: 2008 update Nucleic Acids Res 2008 36 D637 40 10.1093/nar/gkm1001 18000002 PMC2238873

[B23] Uetz P Giot L Cagney G Mansfield T Judson R A comprehensive analysis of protein-protein interactions in Saccharomyces cerevisiae Nature 2000 403 623 627 10.1038/35001009 10688190

[B24] Ito T Chiba T Ozawa R Yoshida M Hattori M Sakaki Y A comprehensive two-hybrid analysis to explore the yeast protein interactome P Natl Acad Sci Usa 2001 98 8 4569 4574 10.1073/pnas.061034498 PMC31875 11283351

[B25] Fromont-Racine M Mayes A Brunet-Simon A Rain J Colley A Dix I Decourty L Joly N Ricard F Beggs J Legrain P Genome-wide protein interaction screens reveal functional networks involving Sm-like proteins Yeast 2000 17 2 95 110 10.1002/1097-0061(20000630)17:2<95::AID-YEA16>3.0.CO;2-H 10900456 PMC2448332

[B26] Gavin A Bösche M Krause R Grandi P Marzioch M Bauer A Schultz J Rick J Michon A Cruciat C Remor M Hofert C Schelder M Brajenovic M Ruffner H Merino A Klein K Hudak M Dickson D Rudi T Gnau V Bauch A Bastuck S Huhse B Leutwein C Heurtier M Copley R Edelmann A Querfurth E Rybin V Drewes G Raida M Bouwmeester T Bork P Seraphin B Kuster B Neubauer G Superti-Furga G Functional organization of the yeast proteome by systematic analysis of protein complexes Nature 2002 415 6868 141 147 10.1038/415141a 11805826

[B27] Ho Y Gruhler A Heilbut A Bader G Moore L Adams S Millar A Taylor P Bennett K Boutilier K Yang L Wolting C Donaldson I Schandorff S Shewnarane J Vo M Taggart J Goudreault M Muskat B Alfarano C Dewar D Lin Z Michalickova K Willems A Sassi H Nielsen P Rasmussen K Andersen J Johansen L Hansen L Jespersen H Podtelejnikov A Nielsen E Crawford J Poulsen V Sorensen B Matthiesen J Hendrickson R Gleeson F Pawson T Moran M Durocher D Mann M Hogue C Figeys D Tyers M Systematic identification of protein complexes in Saccharomyces cerevisiae by mass spectrometry Nature 2002 415 6868 180 183 10.1038/415180a 11805837

[B28] Mering CV Krause R Snel B Cornell M Oliver S Fields S Bork P Comparative assessment of large-scale data sets of protein-protein interactions Nature 2002 417 6887 399 403 10.1038/nature750 12000970

[B29] Mewes H Frishman D Güldener U Mannhaupt G Mayer K Mokrejs M Morgenstern B Münsterkötter M Rudd S Weil B MIPS: a database for genomes and protein sequences Nucleic Acids Research 2002 30 31 34 10.1093/nar/30.1.31 11752246 PMC99165

[B30] Güldener U Münsterkötter M Oesterheld M Pagel P Ruepp A Mewes H Stümpflen V MPact: the MIPS protein interaction resource on yeast Nucleic Acids Research 2006 34 D436 41 10.1093/nar/gkj003 16381906 PMC1347366

[B31] Bader G Donaldson I Wolting C Ouellette B Pawson T Hogue C BIND-The Biomolecular Interaction Network Database Nucleic Acids Research 2001 29 242 245 10.1093/nar/29.1.242 11125103 PMC29820

[B32] Xenarios I Salwínski L Duan X Higney P Kim S Eisenberg D DIP, the Database of Interacting Proteins: a research tool for studying cellular networks of protein interactions Nucleic Acids Research 2002 30 303 305 10.1093/nar/30.1.303 11752321 PMC99070

[B33] Chatr-aryamontri A Ceol A Palazzi L Nardelli G Schneider M Castagnoli L Cesareni G MINT: the Molecular INTeraction database Nucleic Acids Research 2007 35 D572 4 10.1093/nar/gkl950 17135203 PMC1751541

[B34] Gavin A Aloy P Grandi P Krause R Boesche M Marzioch M Rau C Jensen L Bastuck S Dümpelfeld B Edelmann A Heurtier M Hoffman V Hoefert C Klein K Hudak M Michon A Schelder M Schirle M Remor M Rudi T Hooper S Bauer A Bouwmeester T Casari G Drewes G Neubauer G Rick J Kuster B Bork P Russell R Superti-Furga G Proteome survey reveals modularity of the yeast cell machinery Nature 2006 440 7084 631 636 10.1038/nature04532 16429126

[B35] Krogan N Cagney G Yu H Zhong G Guo X Ignatchenko A Li J Pu S Datta N Punna T Peregrín-Alvarez J Tikuisis A Shales M Zhang X Davey M Robinson M Paccanaro A Bray J Sheung A Beattie B Richards D Canadien V Lalev A Mena F Wong P Starostine A Canete M Vlasblom J Wu S Orsi C Collins S Chandran S Haw R Rilstone J Gandi K Thompson N Musso G Onge PS Ghanny S Lam M Butland G Altaf-Ul A Kanaya S Shilatifard A O'shea E Weissman J Ingles C Hughes T Parkinson J Gerstein M Wodak S Emili A Greenblatt J Global landscape of protein complexes in the yeast Saccharomyces cerevisiae Nature 2006 440 7084 637 643 10.1038/nature04670 16554755

[B36] Wootton J Sequences with unusual amino acid compositions Curr opin struct biol 1994 4 413 421 10.1016/S0959-440X(94)90111-2

[B37] Tompa P Csermely P The role of structural disorder in the function of RNA and protein chaperones FASEB J 2004 18 11 1169 1175 10.1096/fj.04-1584rev 15284216

[B38] Sandhu K Intrinsic disorder explains diverse nuclear roles of chromatin remodeling proteins J Mol Recognit 2009 22 1 8 10.1002/jmr.915 18802931

[B39] Kim P Sboner A Xia Y Gerstein M The role of disorder in interaction networks: a structural analysis Molecular Systems Biology 2008 4 179 10.1038/msb.2008.16 18364713 PMC2290937

[B40] Iakoucheva L Radivojac P Brown C O'Connor T Sikes J Obradovic Z Dunker A The importance of intrinsic disorder for protein phosphorylation Nucleic Acids Research 2004 32 3 1037 1049 10.1093/nar/gkh253 14960716 PMC373391

[B41] Reményi A Scholer H Wilmanns M Combinatorial control of gene expression Nat Struct Mol Biol 2004 11 9 812 815 10.1038/nsmb820 15332082

[B42] Pettifer S Thorne D McDermott P Marsh J Villéger A Kell D Attwood T Visualising biological data: a semantic approach to tool and database integration BMC Bioinformatics 2009 10 Suppl 6 S19 10.1186/1471-2105-10-S6-S19 19534744 PMC2697642

[B43] Yook S Oltvai Z Barabási A Functional and topological characterization of protein interaction networks Proteomics 2004 4 4 928 942 10.1002/pmic.200300636 15048975

[B44] Hakes L Pinney J Lovell S Oliver S Robertson D All duplicates are not equal: the difference between small-scale and genome duplication Genome Biol 2007 8 10 R209 10.1186/gb-2007-8-10-r209 17916239 PMC2246283

[B45] Mazurie A http://aurelien.mazurie.oenone.net

[B46] Benjamini Y Hochberg Y Controlling the false discovery rate: a practical and powerful approach to multiple testing Journal of the Royal Statistical Society 1995

