# Supplementary figures and images for: Low-complexity regions within protein sequences have position-dependent roles
Source: BMC Syst Biol. 2010 Apr 13;4:43. doi: 10.1186/1752-0509-4-43 (PMC2873317; doi:10.1186/1752-0509-4-43)

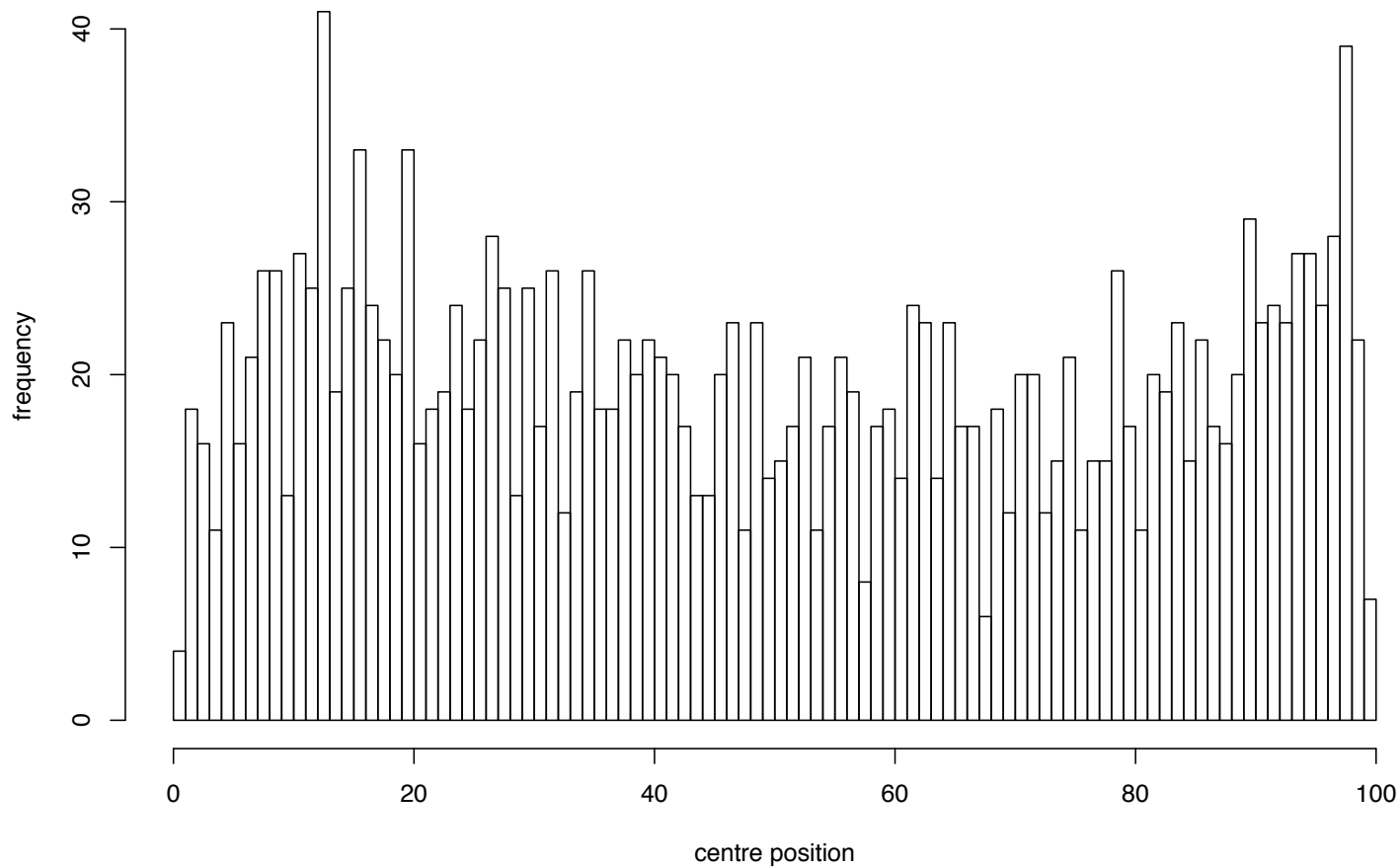

(a)

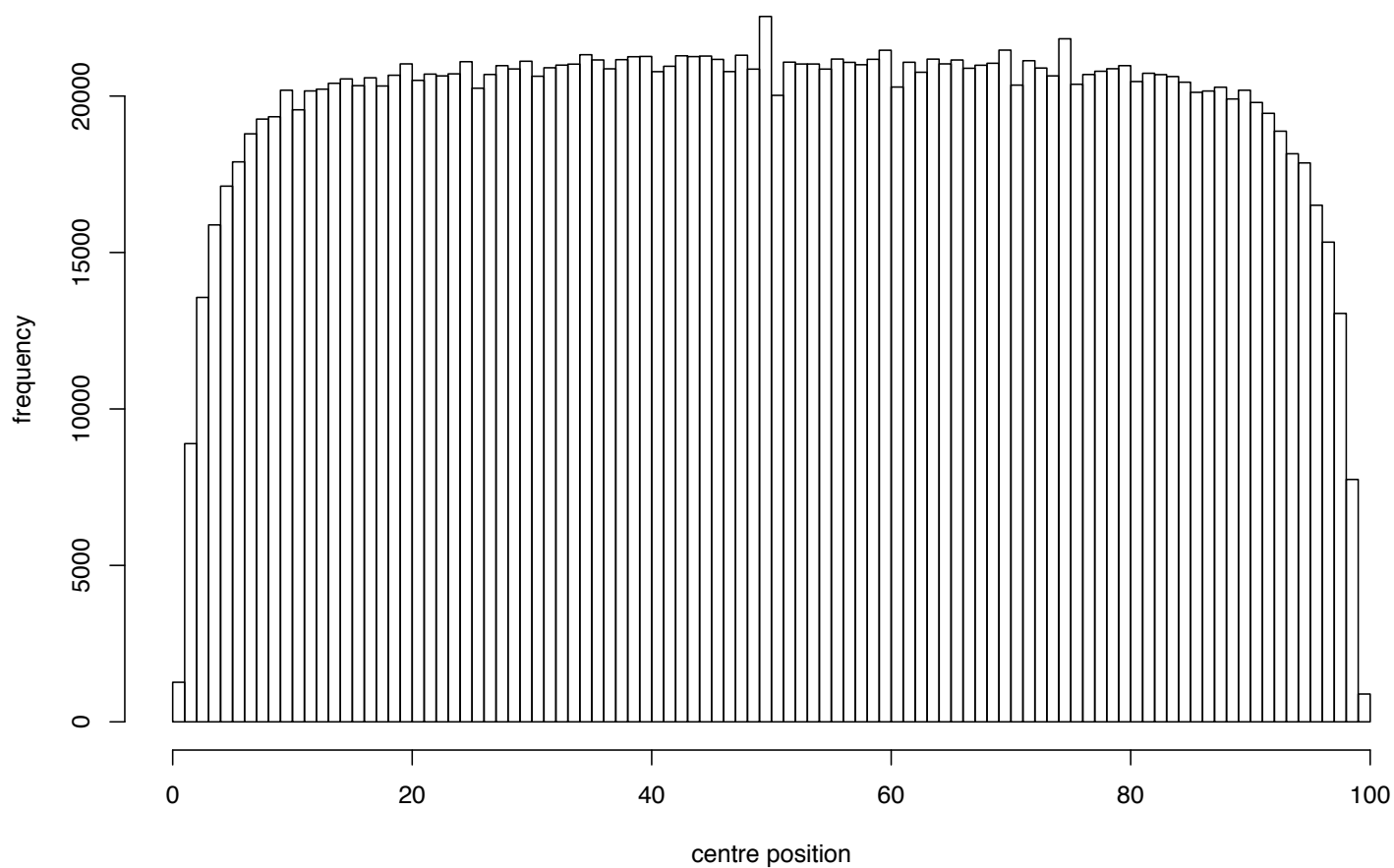

(b)

Supplement: Additional file 1 — Figure S1: LCR distributions in PPI datasets. PPI datasets overlap between the HC, DIPv, FYI and BioGrid datasets, and the distribution of LCRs among them. [file 1752-0509-4-43-S1.PDF]

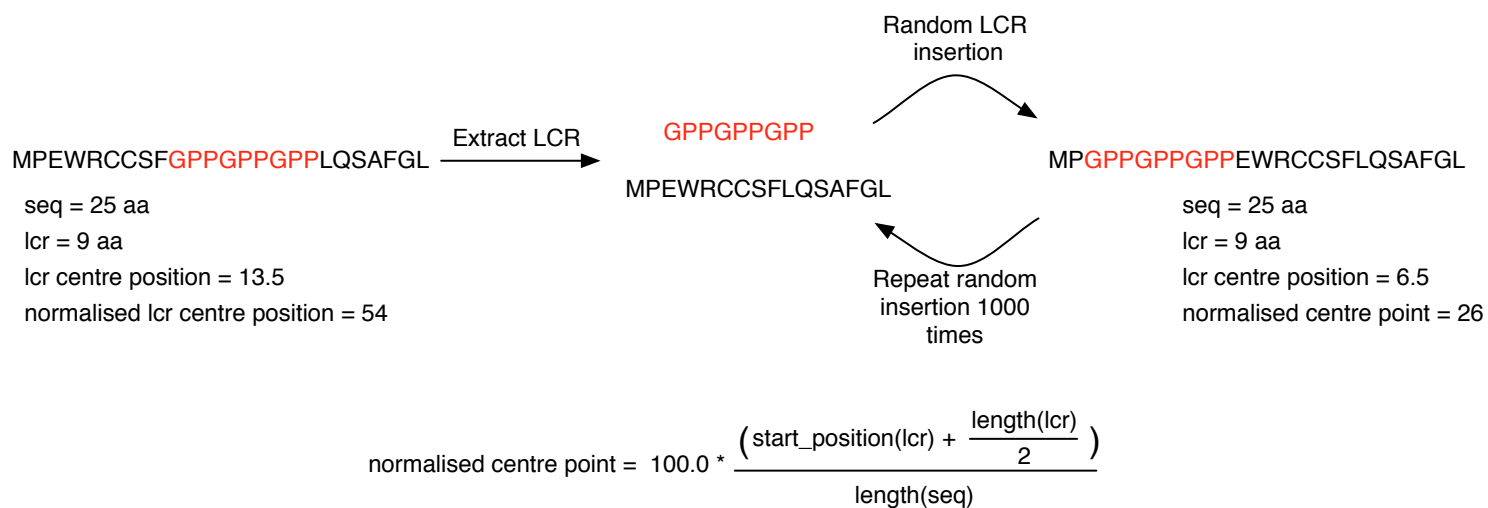

Figure 2:

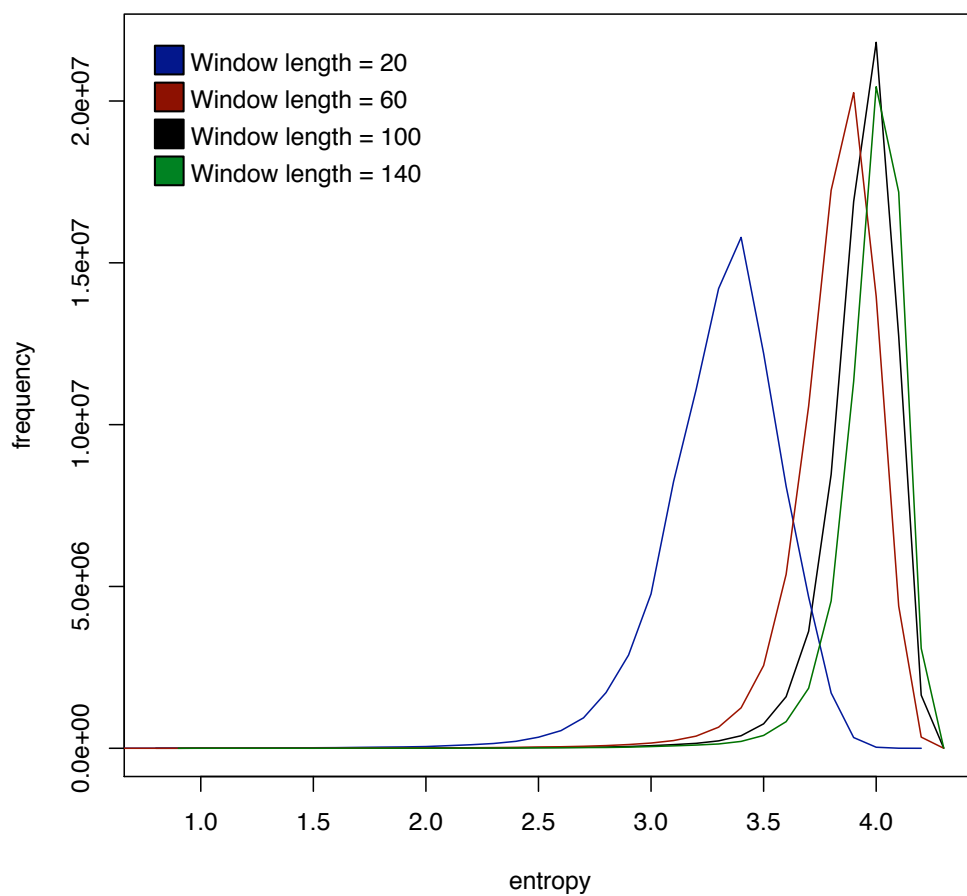

(a) Entropy distributions for different window lengths

Supplement: Additional file 2 — Figure S2: Mean and standard deviation from UniProt entropy distributions. The entropy distributions mean grows asymptotically towards the Hmax value as the window regions increase and sequences within them approach random states. The entropy distributions standard deviation decreases as longer sequences become more homogeneous. [file 1752-0509-4-43-S2.PDF]

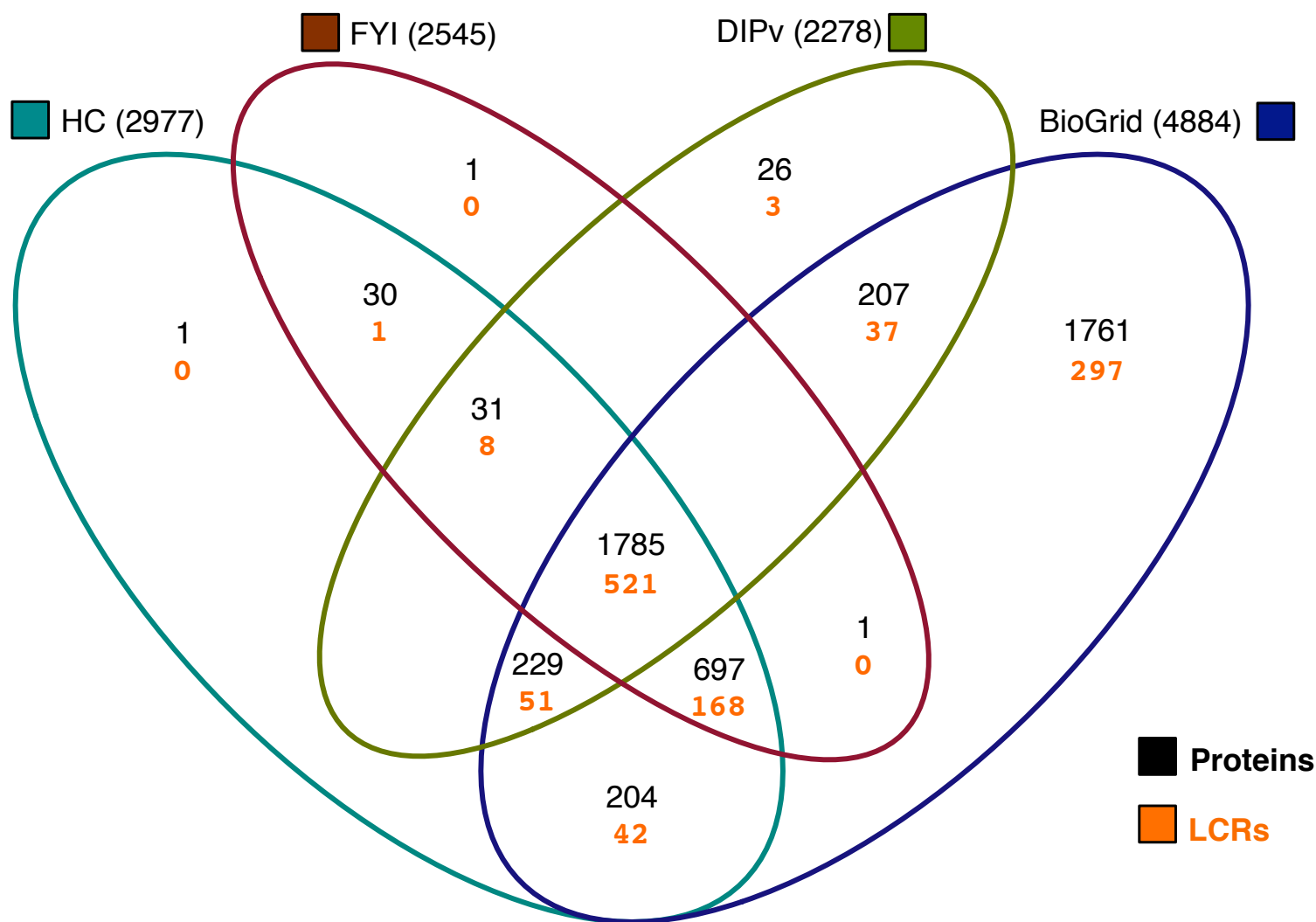

Supplement: Additional file 4 — Figure S4: LCR centre positions distribution. Distributions of LCR centre positions and randomly replaced LCR centre positions. The random distribution extremities show the expected frequency decrease, while the original distribution on top, appears to be enriched with extremity LCRs. [file 1752-0509-4-43-S4.PDF]
