# Supplementary material for: Low-complexity regions within protein sequences have position-dependent roles
Source: BMC Syst Biol. 2010 Apr 13;4:43. doi: 10.1186/1752-0509-4-43 (PMC2873317; doi:10.1186/1752-0509-4-43)
Supplement: Additional file 3 — Figure S3: Computing random LCR positions. Method to compute random LCR positions. The same process is repeated for each LCR in S. cerevisiae: LCRs (shown in red) are extracted from their corresponding protein sequence and re-inserted randomly 1000 times. Each time, the normalised centre position is included into the random distribution. [file 1752-0509-4-43-S3.PDF]

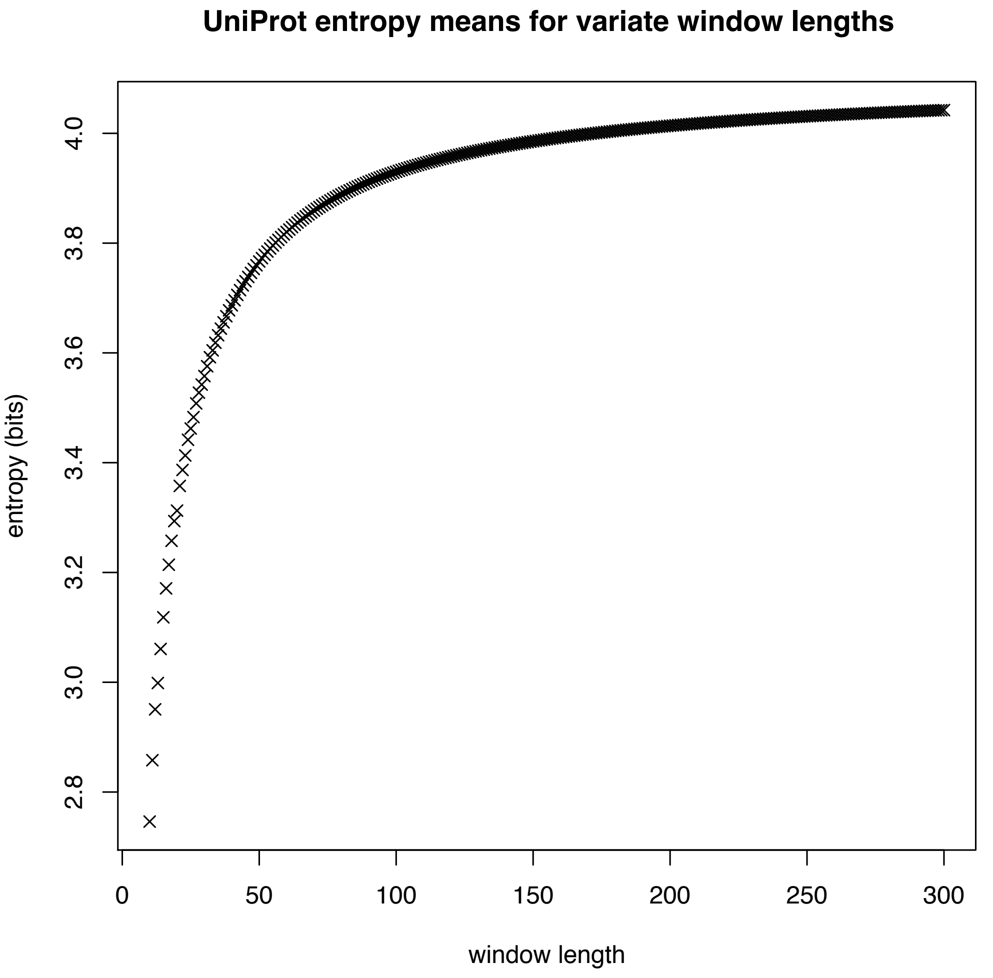

(a) Mean values from UniProt entropy distributions

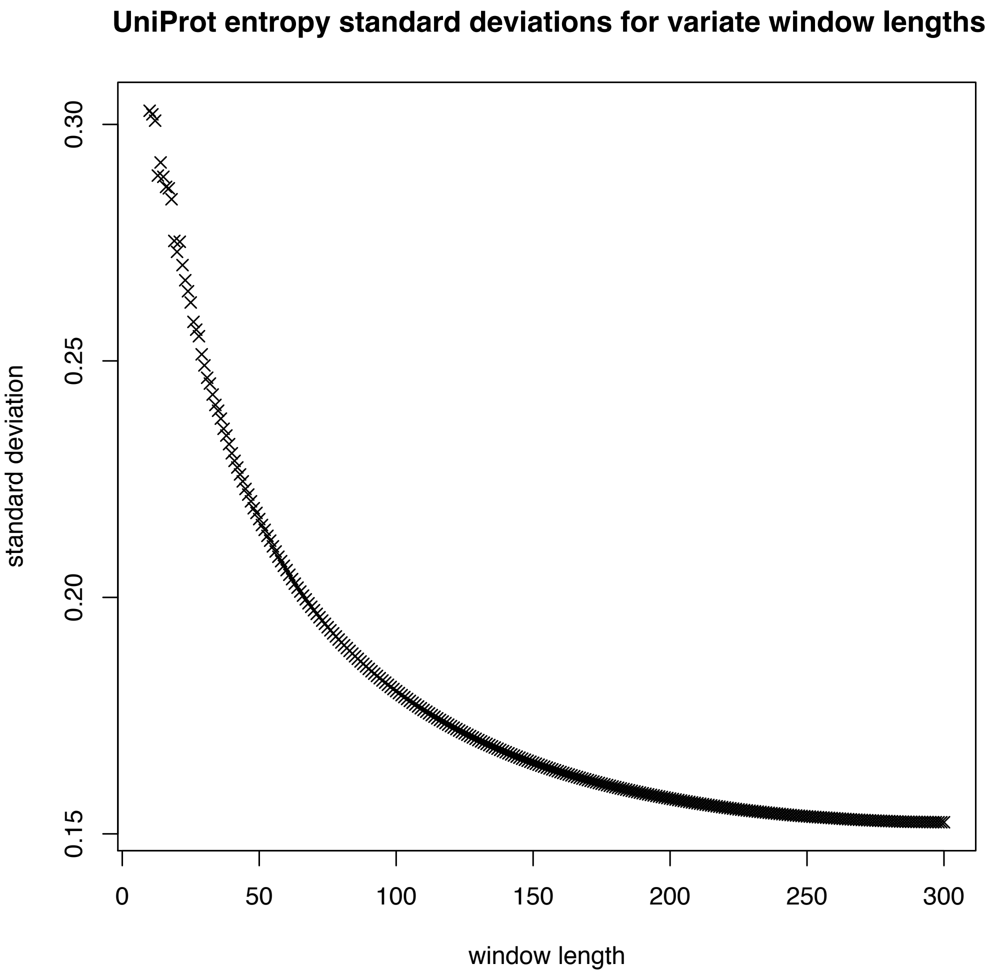

(b) Standard deviations from UniProt entropy distributions
