# Supplementary material for: Low-complexity regions within protein sequences have position-dependent roles
Source: BMC Syst Biol. 2010 Apr 13;4:43. doi: 10.1186/1752-0509-4-43 (PMC2873317; doi:10.1186/1752-0509-4-43)
Supplement: Additional file 5 — Table S1: LCR distributions in PPI datasets. LCRs are approximately equally distributed across the high-confidence datasets (HC, FYI and DIPv). Enrichment is defined as (Observed - Expected)/Expected. [file 1752-0509-4-43-S5.PDF]

Datasets	SGD (6165)	PPI networks G (4884)	HC (2977)	FYI (2545)	DIPv (2278)
Observed LCRs	929	791	548	481	423
Expected LCRs		736.0	448.6	383.5	343.3
Enrichment		7.0%	18.1%	20.3%	18.8%
